# Bridging the mismatch: observing the introduction of new anesthesia technology for a low-resource environment

**DOI:** 10.3389/fmed.2024.1373593

**Published:** 2024-05-02

**Authors:** John Burthorne Sampson, Rahul Koka, Oluwakemi Tomobi, Adaora Chima, Eric Vincent Jackson, Michael Rosen, Michael Koroma, Howard Nelson-Williams, Elizabeth David, Benjamin Lee

**Affiliations:** ^1^Department of Anesthesiology and Critical Care Medicine, Johns Hopkins University School of Medicine, Baltimore, MD, United States; ^2^Department of Anesthesiology, West Virginia University School of Medicine, Morgantown, WV, United States; ^3^Department of Anesthesiology, Cincinnati Children’s Hospital Medical Center, Cincinnati, OH, United States; ^4^Value Institute, Christiana Care Health System, Newark, DE, United States; ^5^Armstrong Institute of Patient Safety and Quality, Johns Hopkins University School of Medicine, Baltimore, MD, United States; ^6^Department of Anaesthesiology, Princess Christian Maternity Hospital, Freetown, Sierra Leone; ^7^Howard University Hospital, Washington, DC, United States; ^8^Walden University, Minneapolis, MN, United States; ^9^Emory University School of Medicine, Atlanta, GA, United States

**Keywords:** Africa, Sierra Leone, global health, anesthesia, low-resource environments

## Abstract

**Objective:**

The objective of this study was to examine the impact of the introduction of the Universal Anaesthesia Machine (UAM), a device designed for use in clinical environments with limited clinical perioperative resources, on the choice of general anesthesia technique and safe anesthesia practice in a tertiary-care hospital in Sierra Leone.

**Methods:**

We introduced an anesthesia machine (UAM) into Connaught Hospital, Freetown, Sierra Leone. We conducted a prospective observational study of anesthesia practice and an examination of perioperative clinical parameters among surgical patients at the hospital to determine the usability of the device, its impact on anesthesia capacity, and changes in general anesthesia technique.

**Findings:**

We observed a shift from the use of ketamine total intravenous anesthesia to inhalational anesthesia. This shift was most demonstrable in anesthesia care for appendectomies and surgical wound management. In 10 of 17 power outages that occurred during inhalational general anesthesia, anesthesia delivery was uninterrupted because inhalational anesthesia was being delivered with the UAM.

**Conclusion:**

Anesthesia technologies tailored to overcome austere environmental conditions can support the delivery of safe anesthesia care while maintaining fidelity to recommended international anesthesia practice standards.

## 1 Introduction

Safe general anesthesia is a drug-induced reversible condition that includes specific behavioral and physiological traits—unconsciousness, amnesia, analgesia, and akinesia—with concomitant stability of the autonomic, cardiovascular, respiratory, and thermoregulatory systems (1). Anesthesia delivery systems are essential intraoperative life support devices used to achieve these goals in surgical patients. Design advances in anesthesia technology have introduced complex features that enhance patient management in high-resource countries but render such devices impractical and unsafe in resource-poor locations that experience frequently interrupted electrical supply, inconsistent access to compressed oxygen (2) and consumables such as carbon dioxide (CO_2_)-absorbing granules (2), and little or no biomedical maintenance support. These constraints hinder the global goal to improve access to safe surgical and anesthesia care as described by the Lancet Commission on Global Surgery and the World Health Assembly in 2015 (3).

The Universal Anaesthesia Machine (UAM^®^) (Gradian Health Systems Inc. New York, United States) was designed to overcome these constraints by providing a source of concentrated oxygen from ambient air, low-resistance vaporizers, and one-way expulsion of CO_2_ in the absence of CO_2_-absorbing granules. The machine could thus enhance the capacity to provide safe and consistent anesthetic care for surgical patients in low-resource conditions.

The objective of this study was to examine the safety and impact of introducing the UAM^®^ into an austere clinical environment with limited perioperative resources. Specifically, we examined the effect of the addition of the UAM^®^ on the practice of total intravenous anesthesia (TIVA) techniques for general anesthesia in a tertiary-care hospital in Sierra Leone. We hypothesized that if local anesthesia providers had access to a reliable, safe, and effective anesthesia delivery system, we would observe a shift among general anesthesia cases from TIVA techniques to increased use of inhalational agents. We also examined the performance of the UAM^®^ in the presence of environmental constraints and reviewed its effects on intraoperative care, postoperative sedation, and analgesic levels after its deployment.

## 2 Methods

### 2.1 Study setting

This study took place at Connaught Hospital in Freetown, Sierra Leone, West Africa. Connaught Hospital is a tertiary-level government hospital with 275 beds that serves as a national referral center, providing all health services except maternal and non-surgical pediatric healthcare. Here, 10–15 cases are performed in the operating rooms.

### 2.2 Ethics

The study approval was obtained from the Sierra Leone Ethics and Scientific Review Committee and our Institutional Review Board. All participants provided consent prior to enrolling in the study. The funding company did not provide any oversight with respect to data review or choice of data collection. They did not see or influence study plans or results, nor did they influence study design, or review outcomes prior to or during abstract and manuscript generation. Safeguards against research influence were incorporated within a memorandum of understanding with the company.

### 2.3 Study design

We conducted a prospective observational study of anesthesia practice among all anesthesia providers at Connaught Hospital (10 nurse anesthetists and 1 physician anesthetist), examining perioperative clinical parameters among a convenience sample of surgical patients. Observed procedures and enrolled patients were those scheduled to receive surgical care on weekdays (M-F) between 8:30 a.m. and 4:00 p.m. and consented to participate in the study, respectively. We designed and piloted the perioperative data collection forms among anesthesia providers at the Johns Hopkins Hospital, Baltimore, Maryland, United States, and subsequently modified these tools in-country to ensure contextually appropriate data variables and minimize data collection ambiguity. Data were collected at the study site over a period of 25 months (June 2012 to July 2014). Baseline clinical anesthesia practice was determined through direct observation of care and documentation of perioperative tasks by anesthesia providers in a preceding observational exercise from June 2012 to February 2013 (pre-UAM^®^ deployment) (4), after which we introduced the UAM^®^ to the hospital. Device deployment was accompanied by a 1-week training course for all anesthesia care providers and biomedical technicians. The training involved basic principles related to the provision of general anesthesia and the use of the UAM. Given the introduction of new UAMs, we worked closely with Gradian Health for a detailed step-by-step walk-through of the use of the UAMs. We continued the observation of anesthesia practice after device introduction for 18 months from February 2013 to August 2014.

We trained seven Sierra Leonean research assistants, including two research nurses, on research methodology, operating room etiquette, and other relevant tasks. These research assistants were also trained to assess clinical care, including vital sign monitoring, and clinical parameters such as pain and consciousness level of patients in the perioperative period. Intraoperative data collection took place from Monday to Friday over a period of 2 years, except on hospital-sanctioned holidays when elective surgical cases were not performed. Research nurses monitored vital signs at designated times in the postoperative period. The Johns Hopkins-based research team (consisting of US-based clinicians with public health expertise) supervised research assistant training and data collection directly until they obtained a kappa statistic of agreement ≥0.7 (5).

Postoperatively, we followed the clinical status of observed cases to hospital discharge or to postoperative day 30 through examination of hospital ward records. The research nurses conducted direct patient clinical assessments for recovery from anesthesia and surgery in the first 72 postoperative hours among a convenience sample of consented patients.

### 2.4 Materials

#### 2.4.1 Anesthesia devices

- The UAM^®^ can be classified as a low-resource optimized anesthesia machine in that it is designed for environments that must contend with power interruptions, compressed oxygen shortages, and biomedical technician limitations. It is an electric anesthesia delivery system with an incorporated high-capacity oxygen concentrator capable of providing a flow of 10 L/min with 95% inspired oxygen delivery to the patient. It utilizes a low-resistance draw-over vaporizer system that permits the combination of continuous flow and/or draw-over anesthesia for varying conditions encountered in resource-challenged, austere environments. Although the UAM^®^ can use compressed cylinder oxygen or pipeline oxygen sources to deliver oxygen and inhaled agents, the low-resistance draw-over vaporizer can function without compressed gases via egress of room air into the system, providing anesthesia delivery to the patient in the absence of compressed gases or electrical power. It is an oxygen sensor that analyzes the inspired oxygen concentration delivered to the patient and displays this on a monitor with a 10-h battery backup. The UAM^®^ model used in this study is designed for spontaneous and/or manually assisted ventilation (with manual bellows, all UAMs^®^ are delivered with an attached multifunction cardiac monitor from a different manufacturer). The UAM^®^ has CE certification for safe use by the European Union, and the system was approved for use by the Sierra Leone Ministry of Health and the IRB for the country. The UAM^®^ was already in use in the United Kingdom at the time of commencing the study. The Johns Hopkins University School of Medicine created an NGO organizational agreement with the authorities in the country and had a memorandum of understanding (MOU) with the authorities in Sierra Leone. The government authorized the UAMs to be used in-country before the study.
- The Compact-3 (manufacturer unknown) is a type of Boyle’s anesthesia machine [a continuous-flow anesthesia machine with five basic elements: (1) a high-pressure supply of gases, (2) pressure gauges on oxygen cylinders, with pressure-reducing valves, (3) flow meters, (4) metal and glass vaporizer bottle for ether, and (5) a breathing system] (6). The Compact-3 was in residence during this study although its utility was intermittent due to frequent mechanical dysfunction.
- A Glostavent^®^ Anaesthesia Machine (Diamedica (United Kingdom) Limited, Grange Hill Industrial Estate, Bratton Fleming, Barnstaple, Devon, EX31 4UH, United Kingdom) was present but malfunctioned (oxygen concentrator and ventilator) and was retired 3 months after the deployment of the UAM^®^.

The Glostavent can also be classified as a low-resource optimized anesthesia machine.

Patient monitoring devices used during data collection included non-invasive blood pressure monitors, Lifebox^®^ pulse oximeters (Lifebox, London, United Kingdom), and electronic thermometers with disposable slips.

### 2.5 Data collection

Using anesthesia data records, research assistants documented anesthesia-related tasks from patient preoperative arrival to postoperative patient handoff. Surgical procedure and patient demographic data were obtained from the operation list or anesthesia/surgery records. Some variables such as American Society of Anesthesiologists (ASA) clinical status scores and elective or emergent status of the case required verbal confirmation from the anesthesia provider.

Anesthesia-related tasks (e.g., airway management), anesthesia technique, vital signs, and electrical and mechanical disturbances in the operating room were recorded. Timestamps for events such as power failures and anesthesia/surgery start and stop times were collected. Appropriate precautions were taken to avoid distracting providers from patient care by suspending questions during active delivery of care and complying with operating room etiquette. In the 30-day postoperative period, data collectors ascertained the admission status of patients using the following categories: a return to the operating theater within the 30-day period, hospital discharge, or death. Research nurses documented vital signs at the following postoperative times: 1, 2, and 4 h and 1, 2, and 3 days. The Wong–Baker Faces Pain Scale was used to assess self-reported postoperative pain scores, and the Richmond Agitation Sedation Scale was used to determine sedation level in the initial 4-h postoperative period (7, 8). We also reviewed operating room logbooks to determine anesthesia caseload and technique, including those performed outside study observation hours.

With an α-error set to 0.05, a modest treatment effect (20% reduction in ketamine TIVA-only cases), and a true failure rate for experimental subjects as 0.4, we needed to enroll 518 experimental subjects and 518 control subjects (for a minimum sample size = 1,036 cases) to be able to reject the null hypothesis that the failure rates for experimental and control subjects are equal with probability (power) of 0.9. Our sample size justification, based on an uncorrected chi-squared test, was appropriate for this study.

Anesthesia records and follow-up data were scanned into data-secure computers, and the information was abstracted for entry into a FileMaker Pro database (FileMaker, Inc., Santa Clara, CA, United States) and subsequently Microsoft Excel (Microsoft Corp., Redmond, WA, United States). Using STATA 12 software (StataCorp LP, College Station, TX, United States), data analyses included frequency distributions, chi-square test, Wilcoxon rank-sum test, two-sample test of proportions, and linear and logistic regression to compare and examine the relationship between variables, using a statistical significance set at a *p-*value of <0.05.

## 3 Results and discussion

According to hospital operating room records (Table 1), 2,764 anesthetic cases were performed between June 2012 and July 2014. Of these, 850 took place before the introduction of the UAM^®^, and 1,917 took place after the introduction of UAM^®^. Table 2 describes the distribution of anesthesia techniques.

**Table 1:** Description of anesthetic cases (Connaught Hospital operating room registry).

Characteristics^a^	Total	Pre-UAM	Post-UAM	Chi-square
	*N* = 2,764 (%)	*N* = 850 (%)	*N* = 1914 (%)	(df) *χ*^2^ *p*-value
Patient demographics				
Female, *n* (%)	839 (30.35)	281 (33.49)	558 (29.2)	χ^2^ (2) 6.324, 0.04
Male, *n* (%)	1910 (69.10)	562 (66.12)	1,348 (70.4)	
Age ≤ 1 year, *n* (%)	150 (5.21)	38 (4.5)	112 (5.9)	χ^2^ (3) 5.0233, 0.17
Age > 1 to <18 years, *n* (%)	600 (21.7)	185 (21.8)	415 (21.7)	
Age 18–65 years, *n* (%)	1798 (65.1)	549 (64.6)	1,249 (65.3)	
Age > 65 years, *n* (%)	216 (7.8)	78 (9.2)	138 (7.2)	
Surgical categories				
ENT, *n* (%)	65 (2.35)	18 (2.1)	47 (2.5)	χ^2^ (9) 14.2, 0.116
General surgery, *n* (%)	1988 (71.9)	629 (74.0)	1,359 (71.0)	
Gynecology, *n* (%)	18 (0.65)	4 (0.5)	14 (0.7)	
Neurosurgery, *n* (%)	37 (1.34)	6 (0.7)	31 (1.62)	
Orthopedics, *n* (%)	259 (9.37)	88 (10.4)	171 (8.9)	
Plastic surgery/facial and reconstructive, *n* (%)	35 (1.26)	10 (1.18)	25 (1.3)	
Thoracic surgery, *n* (%)	3 (0.11)	0 (0)	3 (0.16)	
Urology, *n* (%)	321 (11.61)	89 (10.5)	232 (12.1)	
Procedure not documented, *n* (%)	38 (1.37)	6 (0.7)	32 (1.7)	

ENT, ear, nose, and throat; UAM, Universal Anaesthesia Machine.

a Categories may not add up to 100% due to undocumented cases.

**Table 2:** Description of all performed anesthesia techniques (operating room registry).

	Total	PreUAM	Post-UAM	Z	*p* value
	*n* = 2,764	*n* = 850 (%)	*n* = 1914 (%)		
Total general anesthesia (GA) cases	1,610	475 (55.9)	1,135 (59.3)	−1.67	0.094
Scheduled GA	1,551	445 (52.4)	1,106 (57.8)	−2.64	0.008
Spinal converted to GA	49	25 (2.94)	24 (1.25)	3.11	0.002
Local converted to GA	10	5 (0.59)	5 (0.26)	1.32	0.186
Anesthesia agents used					
Inhalational anesthesia	890	234 (27.5)	656 (34.3)	−3.53	<0.001
Halothane	890	234 (27.8)	656 (34.3)	−3.53	<0.001
Total intravenous anesthesia	719	240 (28.2)	479 (25.0)	1.77	0.077
Ketamine	658	225 (26.5)	433 (22.6)	2.22	0.026
Propofol	26	13 (1.5)	13 (0.07)	2.01	0.045
Thiopental	31	2 (0.2)	29 (1.5)	−3.02	0.002
Regional techniques					
Spinal anesthesia	821	242 (28.5)	579 (30.3)	−0.96	0.339
Local anesthesia	299	108 (12.7)	191 (10)	2.13	0.033
Anesthesia technique not documented	6	1 (0.1)	5 (0.3)		

A two-sample test of proportions was used. UAM, Universal Anaesthesia Machine.

We reviewed all general anesthesia techniques performed in pre-and post-UAM^®^ study phases and, after controlling for age and surgical categories, observed a 1.6-fold increase (*p* = 0.001, 95% CI [1.19–2.14]) in the odds of inhalational anesthesia administration compared to TIVA, in the post-UAM^®^ period. This shift from TIVA (predominantly ketamine) to inhalational anesthesia was most demonstrable in anesthesia care for patients undergoing appendectomies and surgical wound management (Table 3).

**Table 3:** General anesthesia use among the 10 most common surgical cases performed (operating room registry).

Procedure	Total performed in pre- and post-period		Pre-UAM				Post-UAM			Odds of INH as the choice of GA, pre-*vs* post UAM		
		Total pre-UAM	GA	INH, *n* (%)	TIVA, *n* (%)	Total post-UAM	GA	INH, *n* (%)	TIVA, *n* (%)	OR	95% CI	*p*-value
Hernia repair	918	261	107	77 (72)	30 (28)	657	289	207 (71.6)	82 (28.4)	0.98	0.58–1.65	0.947
Appendectomy	213	62	53	18 (34)	35 (66)	151	112	61 (54.5)	51 (45.5)	2.33	1.12–4.89	0.014
Laparotomy	205	62	62	30 (48.4)	32 (51.6)	143	139	71 (51.1)	68 (48.9)	1.11	0.59–2.12	0.724
Prostatectomy	108	40	6	2 (33.3)	4 (66.7)	68	0	0	0	NA	NA	NA
Wound debridement/exploration	103	24	18	2 (11.1)	16 (88.9)	79	64	25 (39.1)	39 (60.9)	5.13	1.04–48.99	0.026
Lumpectomy	87	45	29	12 (41.4)	17 (58.6)	42	26	13 (50.0)	13 (50.0)	1.42	0.43–4.7	0.522
Amputation	81	26	6	2 (33.3)	4 (66.7)	55	12	5 (41.7)	7 (58.3)	1.43	0.13–21.41	0.732
Urethral calibration	80	15	15	1 (6.7)	14 (93.3)	65	57	4 (7.0)	53 (93.0)	1.06	0.09–55.7	0.962
Urethral repair	78	21	5	1(20)	4(80)	57	8	6(75.0)	2 (25.0)	12	0.54–686.48	0.053
Hydrocelectomy	76	25	10	4 (40)	6(60)	51	19	14 (73.7)	5 (26.3)	4.2	0.64–28.96	0.076

GA, general anesthesia; INH, inhalational anesthesia; TIVA, total intravenous anesthesia; OR, odds ratio; CI, confidence interval; NA, not available.

### 3.1 Directly observed anesthesia cases

In the post-UAM^®^ phase, we observed 870 cases of perioperative anesthesia care (Table 4), 45.5% of all cases performed. In total, 20 of these cases were described as emergent by local providers.

**Table 4:** Anesthesia cases/techniques performed on directly observed cases (directly observed by the team).

Anesthesia cases	Total	Pre-UAM	Post-UAM
	*n* = 1,374	*n* = 504 (%)	*n* = 870 (%)
ASA classification			
ASA I	867	340 (67.5)	527 (60.6)
ASA II	405	131 (26)	274 (31.5)
ASA III	31	10 (2.0)	21 (2.4)
ASA IV	2	2 (0.4)	0 (0)
ASA unknown	69	21 (4.2)	48 (5.5)
Anesthesia technique			
General anesthesia^a^	845	287 (56.9)	558 (64.1)
Inhalational	676	205 (40.7)	471 (54.1)
TIVA	169	82 (16.3)	87 (10)
Regional anesthesia	493	193 (38.3)	310 (35.6)
Spinal	364	138 (27.4)	226 (26)
Local	127	55 (10.9)	84 (9.7)
Monitored anesthesia care	25	24 (4.8)	1 (0.1)
Intraoperative change in anesthesia technique	76	29 (5.8)	47 (5.4)
Regional converted to general anesthesia	71	28 (5.6)	43 (4.9)
Airway management			
Endotracheal intubation	391	112 (22.2)	279 (32.1)
Laryngeal mask airway	28	11 (2.2)	17 (2.0)
Oropharyngeal airway +/− facemask	297	117 (23.2)	180 (20.7)
Facemask	387	212 (42.1)	175 (20.1)
None	378	159 (31.5)	219 (25.2)

ASA, American Society of Anesthesiologists; TIVA, total intravenous anesthesia; UAM, Universal Anaesthesia Machine.

a Includes regional anesthesia cases converted to general anesthesia.

### 3.2 Intraoperative period

Among regional cases converted to general anesthesia (Table 4), inhalational agents were utilized 54% of the time. Providers monitored blood pressure in 612 (70.3%) patients, pulse oximetry in 827 (95.1%), electrocardiography in 30 (3.5%), temperature in 23 (2.7%), and capnography in 10 (1.15%). Blood pressure was more likely to be measured in patients older than 18 years of age, χ^2^ (2, *N* = 870) *p* < 0.001.

We observed 343 incidents of intraoperative tachycardia (heart rate > 100 beats/min for ≥10 min in patients >12 years). The proportion of patients who experienced tachycardia did not differ among patients who received inhalational anesthesia from the UAM^®^ and those whose anesthesia was delivered by another machine, z = 1.96, *p* = 0.05.

The portable free-standing oxygen concentrator onsite had a maximum delivery capacity of 5 L/min and had been improvised to connect to the Compact-3 anesthesia machine. All observations where oxygen delivery flow rates were greater than 5 L/min occurred in cases involving the UAM^®^ or an oxygen tank. Compressed cylinder oxygen was unavailable for >50% of the duration of the total observational period, and, when available, there was no reliable method to confirm the oxygen concentration in the cylinder.

The UAM^®^ was used to deliver inhalational anesthesia in 287 cases, and for supplemental oxygen delivery in 38 cases that did not involve the use of inhalational anesthesia. The choice of anesthesia machine did not affect the duration of anesthesia or surgical care (Table 5).

**Table 5:** Perioperative care duration (minutes) among inhalational anesthesia cases administered by UAM and non-UAM machines.

	Inhalational UAM			Inhalational non-UAM					
	M	SD	CI	M	SD	CI	df	*t*-test	*p*-value
Anesthesia duration^a^	61.6	36.4	57.4–65.9	59.4	39.4	53.7–65.2	466	−0.62	0.53
Anesthesia care duration^b^	71	39.3	66.4–75.6	68.4	41	62.4–74.4	466	−0.68	0.49
Emergence time^c^	10.2	11.5	8.8–11.5	10.4	11.5	8.7–12.03	466	0.16	0.87
Surgery duration^d^	41.3	30.7	37.7–44.9	38.3	29.5	34.02–42.7	466	−1.03	0.31

UAM, Universal Anaesthesia Machine; M, mean; SD, standard deviation; CI, confidence interval; df, degrees of freedom.

a Anesthesia duration: commencement of induction to completion of surgery (wound dressing applied).

b Anesthesia care duration: Time from first contact with the patient to time when the patient is handed off for transfer to the ward or immediate postoperative discharge.

c Emergence time: Time from surgery end to when the patient is handed off for transfer to the ward or immediate postoperative discharge.

d Surgery time: Time from incision to wound dressing application or surgeon communicates completion.

We recorded 27 instances of power outages ranging from 1 min to 90 min in duration. In total, 17 of these occurred during inhalational anesthesia delivery, of which 10 were administered with the UAM^®^. There were no interruptions in the latter as the UAM^®^ immediately reverted to room air (with inhalational anesthetic), while ventilation in other cases was continued with an Ambu bag^®^ (without inhalational anesthetic). There were two occasions of reported anesthesia equipment-related malfunction. On investigation, neither originated from the machine. One incident was a power surge, which damaged fuses in the UAM^®^ as a result of surge protector removal prior to the event.

Biomedical technicians at Connaught Hospital replaced the fuses within hours of discovery, and the machine returned to full service. In the second event, the oxygen monitor displayed an alarm for the replacement of the oxygen sensor. This was initially mistaken for low oxygen concentration readings and occurred 11.5 months after the installation of the machine. The manufacturer recommends replacing the oxygen sensor after 12 months.

### 3.3 Postoperative outcomes and mortality

A Wilcoxon rank-sum test revealed a difference in hours 2 and 4 pain scores between TIVA and inhalational anesthesia cases (Table 6).

**Table 6:** Pain and level of consciousness scores among inhalational cases (by type of anesthesia machine) and general anesthesia cases 1–4 h postoperatively.

Postoperative time	Type of anesthesia machine	*N*	Mean	Wilcoxon rank-sum test		Type of GA	*N*	Mean	Wilcoxon rank-sum test	
				*Z*	*p*-value				*Z*	*p*-value
Postoperative pain										
1 h	Non-UAM	110	0.77	0.28	0.78	Inhalational	304	0.79	0.94	0.35
	UAM	194	0.8			TIVA	65	0.85		
2 h	Non-UAM	108	1.45	0.43	0.67	Inhalational	289	1.4	2.06	0.039
	UAM	181	1.37			TIVA	65	1.92		
4 h	Non-UAM	71	2.45	−0.87	0.38	Inhalational	198	2.58	2.76	0.006
	UAM	127	2.65			TIVA	51	3.41		
Level of consciousness										
1 h	Non-UAM	143	−0.53	0.20	0.84	Inhalational	366	−0.56	0.16	0.87
	UAM	223	−0.56			TIVA	71	−0.07		
2 h	Non-UAM	135	−0.27	0.26	0.79	Inhalational	342	−0.29	−0.11	0.91
	UAM	207	−0.30			TIVA	71	−0.35		
4 h	Non-UAM	94	−0.03	−0.23	0.82	Inhalational	256	−0.02	−1.24	0.22
	UAM	162	−0.01			TIVA	52	−0.12		

UAM, Universal Anaesthesia Machine; GA, general anesthesia; TIVA, total intravenous anesthesia.

Linear regression showed this to be significant at only hour 4, with pain scores lower in inhalational cases by −0.83, 95% CI [−1.37 to 0.29], *p* = 0.003. The rate of consciousness recovery did not differ between the two groups in hours 1, 2, or 4 postoperatively. Anesthesia machine differences used did not demonstrate a change in postoperative pain or level of consciousness scores at the 1-, 2-, or 4-h monitoring times (Table 6). In total, 30-day postoperative mortality among observed cases was 2.3% (20 patients), with a risk ratio of 1.66, 95% CI [0.7–3.9], *p* = 0.24, compared to the pre-UAM^®^ period. We were unable to determine the postoperative status of 21 patients during the study period. These were either cases that were canceled mid-procedure or cases that we were unable to follow for the designated follow-up period owing to the 2014 Ebola Viral Hemorrhagic outbreak. Among inhalational anesthesia cases, we found no relation between mortality and the type of anesthesia machine used, χ^2^(1, *N* = 454) = 0.16, *p* = 0.691.

## 4 Discussion

In 2008, the World Health Organization commenced the Global Initiative on Health Technologies to promote the design of innovative technologies adapted for use in resource-limited settings, among other goals. This initiative was borne out of a recognition of the mismatch between available health technology and health infrastructure in many low-resource locations (9, 10). A 2011 study of medical devices in developing countries revealed that, on average, 38.3% of medical technologies in such locations were out of service (8). These findings have been attributed to a lack of appropriate training and infrastructure as well as technological mismanagement (11, 12). The oxygen sensors continue to be a problem. More recent studies involving the UAM aimed to examine simulation methodologies to adopt the use of the UAM (13–15). In the case of anesthesia technology, other factors that may be responsible for the premature retirement of devices include unreliable electricity, absence of compressed gases, insufficient biomedical expertise for maintenance, and ill-suited inhalational agent vaporizers. Although three anesthesia machines were used in varying degrees during the project, six anesthesia machines were physically present in the operating suite areas (Table 6).

As described above, we identified a significant increase in the use of inhalational anesthetic agents following the installation of the UAM^®^. Among failed regional anesthesia cases, the proportion converted to inhalational anesthesia relative to TIVA was significantly higher after the UAM^®^ was introduced into the environment, *z* = 4.56, *p* ≤ 0.001.

The routine practice prior to the UAM^®^ often included holding parts of the anesthesia machine together. In the absence of active maintenance agreements with anesthesia machine manufacturers, these machines were repaired by biomedical technicians and anesthesia staff through improvisation and inventive use of available materials or parts from other machines, which required securing them together so that they do not fall apart. Other challenges included unreliable oxygen delivery to the patient.

While power outages are relatively rare events, when they occur, then there is an increased risk to patients and barriers to the safe use of anesthesia. These events can be stressful and challenging, potentially leading to avoidance of the use of general anesthesia. With a system specifically designed for use in constrained environments, the UAM^®^ allows for more consistently reliable delivery of general anesthesia and possibly more comfort for providers to utilize general anesthesia in these challenging environments.

During the study period, the facility experienced a number of unforeseen challenges that had adverse effects on surgical productivity. These events included shortages of inhalational anesthetics, periodic interruptions to compressed oxygen production and availability, autoclave breakdowns, and a shutdown of the operating room as a result of flooding from extreme weather. Although power outages were frequent, these were mitigated by the presence of a functional generator. Some of these impediments to surgical delivery could not be ameliorated by the UAM^®^, whereas some were specifically overcome by qualities of the UAM^®^. These include:

- efficient oxygen concentrator: able to administer 10 L/min of 95% oxygen, advantageous during the study period as compressed oxygen was unavailable >50% of the time.
- an oxygen analyzer: reliably measures the percentage of oxygen available to the patient by the oxygen concentrator. Before the study, we had been unable to confirm the concentration of oxygen in cylinders because the hospital lacked an oxygen analyzer. This monitor is powered by a trickle charge from the main power supply. This safety feature is critical to monitoring inspired oxygen content, especially during simultaneous power outages and compressed oxygen shortages.
- a low-pressure vaporizer: It enables continued inhalational anesthesia delivery during the simultaneous absence of compressed oxygen and electricity. In these events, the UAM^®^ reverts to room air draw-over anesthetic mode, sustaining the patient at 21% oxygen. There were 10 incidents of power outages during inhalational anesthesia delivery with the UAM^®^.
- a halothane vaporizer: Although halothane has been largely replaced by isoflurane in high-income countries because of related side effects, it is still widely used in many African nations owing to its low cost (16, 17). The UAM^®^ is outfitted with two detachable vaporizers: one each for halothane and isoflurane. Halothane and ether were the only available inhalational anesthetics.
- a one-way valve system: It prevents rebreathing and provides unidirectional gas flow. In this environment, CO_2_-absorbing granules are often not available or replaced. End-tidal CO_2_ (ETCO_2_) levels are not routinely measured, and hypercapnia may go undiagnosed with rebreathing systems. Although two capnography monitors were available in the operating suites, ETCO_2_ was rarely monitored as described in the Results section. The manufacturer recommends the use of passive scavenging of exhaust gases from the system. We did not observe adherence to these guidelines. Reasons for not using capnography included its unavailability in Sierra Leone at the time, the fact that capnography was not part of the UAM monitoring package, and, even when capnography monitors were introduced, the nurse anesthetists did not adopt its use because they were never formally trained in it, and the leadership was reluctant due to not having a good knowledge base for using it.

### 4.1 Implications

Among all cases, we identified an increase in the proportion of general anesthetics performed, with the greater percentage being inhalational anesthesia, rather than ketamine TIVA. In debrief interviews, anesthesia providers described the UAM^®^ as “simple,” “convenient,” and “straightforward.” Multiple users recommended the inclusion of an automated ventilator mode to ease the workload of manual ventilation during long cases. Subsequent UAM^®^ models designed since the conclusion of the study include a mechanical ventilator.

### 4.2 Perioperative outcomes

We did not detect a difference in the occurrence of adverse events between UAM^®^ and non-UAM^®^ cases. With respect to postoperative pain, we identified a slight decrease in pain scores at hour 4 among inhalational cases compared to TIVA. There were no differences in analgesic administration. This fact is noteworthy; as halothane has minimal to no analgesic properties, it was often co-administered with boluses of intravenous anesthetics or narcotic analgesics, especially during anesthetic induction. Such co-administration occurred in 342 cases. It is possible that more reliable depth of anesthesia and quality general anesthesia may have led to less noxious stimulation during the surgery and may have led to pre-emptive analgesia, and thus difference in pain scores.

### 4.3 Limitations

While the training program can be viewed as a confounding and contributing factor in the outcome of the study, from its inception, the study design made *a priori* assumptions that training on clinical use and maintenance were essential elements to the acceptance and use of the UAM in common clinical practice. The training program does not overcome the obstacles of oxygen availability, stable electricity, and maintenance ease. However, it would be unethical to deploy novel technology in the absence of relevant training.

## 5 Conclusion

Anesthesia technologies tailored to overcome austere environmental conditions have the ability to deliver safe anesthesia care while maintaining fidelity to recommended international anesthesia practice standards. In this study, we observed the *in situ* use of a low-resource optimized anesthesia machine, the Universal Anaesthesia Machine. In an environment with multiple unfavorable conditions, we were able to determine that the UAM^®^ provided efficient and reliable anesthetic delivery without adverse outcomes. Since UAM^®^ introduction at Connaught Hospital, a shift in ketamine-TIVA anesthetic administration to inhalational general anesthesia was observed. While there is no basis for identifying one anesthetic technique as superior to another, there are advantages to having options for different types of anesthesia for different types of cases and different types of patients.

The UAM^®^ functioned without any significant mechanical problems and provided a reliable source of oxygen via the concentrator with an oxygen sensor/monitor to ensure adequate oxygen delivery during surgery. The use of devices that are designed to function in challenging austere environments, require minimal maintenance, and utilize local sources of replacement parts, combined with training of providers and technicians, should theoretically provide reliable, safe, and efficient care in these settings. Currently, there are two UAMs at Connaught that are frequently used. The maintenance team has been able to fix any problems that arise. Capnography is also being used. Pursuant to the 2015 World Health Assembly’s Resolution on Surgery and Anesthesia Care (3), it is imperative to examine the technological resources available to perioperative providers working in constrained conditions and strive to engineer appropriate technology for safe perioperative care in their environments.

## Data availability statement

The original contributions presented in the study are included in the article/supplementary material, further inquiries can be directed to the corresponding author.

## Ethics statement

The studies involving humans were approved by Johns Hopkins Institutional Review Board; Sierra Leone Ethics and Scientific Review committee. The studies were conducted in accordance with the local legislation and institutional requirements. The participants provided their written informed consent to participate in this study.

## Author contributions

JS: Writing – original draft, Writing – review & editing, Investigation, Methodology. RK: Writing – review & editing, Formal analysis, Investigation. OT: Writing – review & editing. AC: Data curation, Writing – review & editing, Methodology. EJ: Writing – review & editing, Investigation, Methodology. MR: Writing – review & editing, Investigation. MK: Writing – review & editing, Methodology, Investigation. HN-W: Writing – review & editing. ED: Writing – review & editing. BL: Funding acquisition, Investigation, Methodology, Writing – review & editing.
